# Economic Evaluation of Obesity Prevention in Early Childhood: Methods, Limitations and Recommendations

**DOI:** 10.3390/ijerph13090911

**Published:** 2016-09-13

**Authors:** Nora Döring, Susanne Mayer, Finn Rasmussen, Diana Sonntag

**Affiliations:** 1 Child and Adolescent Public Health Epidemiology, Department of Public Health Sciences, Karolinska Institutet, Stockholm 17177, Sweden; nora.doering@ki.se (N.D.); finn.rasmussen@ki.se (F.R.); 2 Department of Health Economics, Centre for Public Health, Medical University of Vienna, Vienna 1090, Austria; 3 Health Care Services, Stockholm County Council, Centre for Epidemiology and Community Medicine, Stockholm 17129, Sweden; 4 Mannheim Institute of Public Health, Social Prevention and Medicine (MIPH), Medical Faculty of Heidelberg University, Mannheim 68167, Germany; mail@diana-sonntag.eu; 5 Department of Health Science, University of York, Heslington, York YO10 5DD, UK

**Keywords:** cost effectiveness, early childhood, obesity, prevention, methods

## Abstract

Despite methodological advances in the field of economic evaluations of interventions, economic evaluations of obesity prevention programmes in early childhood are seldom conducted. The aim of the present study was to explore existing methods and applications of economic evaluations, examining their limitations and making recommendations for future cost-effectiveness assessments. A systematic literature search was conducted using PubMed, Cochrane Library, the British National Health Service Economic Evaluation Databases and EconLit. Eligible studies included trial-based or simulation-based cost-effectiveness analyses of obesity prevention programmes targeting preschool children and/or their parents. The quality of included studies was assessed. Of the six studies included, five were intervention studies and one was based on a simulation approach conducted on secondary data. We identified three main conceptual and methodological limitations of their economic evaluations: Insufficient conceptual approach considering the complexity of childhood obesity, inadequate measurement of effects of interventions, and lack of valid instruments to measure child-related quality of life and costs. Despite the need for economic evaluations of obesity prevention programmes in early childhood, only a few studies of varying quality have been conducted. Moreover, due to methodological and conceptual weaknesses, they offer only limited information for policy makers and intervention providers. We elaborate reasons for the limitations of these studies and offer guidance for designing better economic evaluations of early obesity prevention.

## 1. Introduction

Overweight and especially obesity are a growing public health concern worldwide. While the target group of obesity prevention has long been mainly adults and school children, there has also been an increased awareness over the last decade of the need to tackle overweight and obesity among preschool children. Children with overweight and/or obesity not only run an increased risk of becoming obese as adults [1] but are also likely to develop early signs and symptoms of co-morbidities, insulin resistance [2] and hypertension [3,4] during childhood. Eating and physical activity habits are established early in life and become less malleable in later life [5].

In addition to health-related consequences and psychosocial problems [6], obesity implies significant economic consequences through direct costs [7]. According to recent estimates for the European Union, the annual direct costs of obesity amount to 7% of the national health budgets [8]. These costs impose a large burden on the already pressured health care sector and challenge the sustainability of health care systems. Moreover, obesity and its related co-morbidities can lead to tremendous societal consequences due to indirect costs. Productivity losses associated with overweight and obesity (sick leave, disability pension, death before retirement or other types of exclusion from the labour market, e.g., stigmatisation) are estimated to even exceed direct costs [9,10]. Furthermore, lower educational attainment among children with overweight and obesity may reduce their chances on the labour market. Due to the wide range of health, social and economic consequences, policy makers and intervention providers are in great need of evidence-based prevention programmes.

For all the above reasons the prevention of childhood obesity is a key element in current public health strategies. Indeed, there seems to be consensus that behavioural interventions initiated early in childhood rather than later in life may have the greatest preventive effect [11]. However, despite an ever-growing number of intervention studies for that age group, the evidence on what works and what does not work is far from conclusive [11,12,13,14]. However, strong evidence for effectiveness alone is insufficient for policy makers responsible for resource allocation: A critical and timely analysis of the cost-effectiveness of new childhood obesity programmes is essential to ensure an optimal and sustainable decision process.

Despite methodological advances in economic evaluations and growing evidence in the field of childhood obesity prevention, there have been only a few studies analysing the cost-effectiveness of prevention interventions targeting preschool children. In 2009, Carter et al. [15] reviewed existing childhood obesity interventions in Australia and assessed their cost-effectiveness. The primary aim of their review was to provide a general overview of various interventions and associated costs. In line with the Assessing Cost-Effectiveness (ACE) obesity approach [16], all their economic evaluations followed the same methodology, which allowed a comparison between interventions. The authors compared results from a variety of Australian intervention studies, including behavioural, pharmaceutical and surgical interventions. Their definition of childhood ranged from preschool children (<6 years of age) to adolescents (14–19 years of age). However, these authors did not provide an overview and a critical assessment of existing methods. A similar approach was applied in the review by Gortmaker et al. [17], in which the ACE approach was adapted to the U.S. setting. Purposive sampling of intervention studies was applied to represent a “broad range of national scalable interventions”, including, in the end, four intervention studies focusing on (1) excess taxes of sugar-sweetened beverages; (2) elimination of tax deductibility of advertising costs; (3) state policy of moderate to vigorous physical activity during >50% of time in physical education classes; (4) state policy of a healthier early childhood educational setting.

Our article aims to systematically review existing methods and applications of economic evaluations in the field of early childhood obesity prevention. We shed light on methodological and conceptual limitations and offer recommendations on how to design better economic evaluations.

## 2. Materials and Methods

### 2.1. Search Strategy

Following the Centre for Reviews and Dissemination guidance for undertaking reviews in health care [18], we conducted a systematic literature review between September 2015 and November 2015 using the main electronic databases for health sciences and health economic evaluations, including PubMed, Cochrane Library, the British National Health Service Economic Evaluation Databases (NHS EED) and EconLit. Studies published between January 2004 and November 2015 were considered for inclusion. The year 2004 was chosen since it marks the recognition of the so-called obesity epidemic and the first WHO action plan against obesity [19]. Literature published prior to this is unlikely to be relevant to current circumstances, especially given the developments in economic evaluations over the last decade. An independent librarian applied a search strategy for Medline, which was based on medical subject headings (MeSH) terms and text words of key articles that we identified beforehand. The search string was pretested and modified to strengthen the sensitivity and specificity of the search. The following string was used for the final search:

((“Child, Preschool” (mh) OR Infant (mh)) AND (Overweight/therapy (mh) OR Overweight/psychology (mh) OR “Pediatric Obesity/therapy” (mh) OR “Pediatric Obesity/psychology” (mh)) AND (“Primary Health Care” (mh) OR “Child Health Services” (mh) OR “Primary Prevention” (mh: Noexp) OR “Health Education” (mh) OR “Health Promotion” (mh) OR “Health Knowledge, Attitudes, Practice” (mh) OR “Delivery of Health Care” (mh) OR Counselling (mh) OR “Child Guidance” (mh) OR “Child Rearing” (mh: Noexp) OR “Family Practice” (mh) OR “Parent-Child Relations” (mh) OR Parenting (mh) OR Parents (mh) OR “Food Habits” (mh) OR “Food Preferences” (mh)) AND (“English” (Language) OR “German” (Language)) AND (hasabstract) NOT (“Qualitative Research” (mh) OR “Prevalence” (mh) OR “Socioeconomic Factors” (mh) OR “Ethnic Groups” (mh))) AND (Cost* OR Economic*). 

### 2.2. Inclusion and Exclusion Criteria

Eligible were studies (Table 1) that included either a trial-based cost-effectiveness analysis or a simulation-based cost-effectiveness analysis of an obesity prevention targeting preschool children and/or their parents. Since the review focuses on the cost-effectiveness of early childhood obesity prevention programmes, age was restricted to <6 years. When the age range exceeded the limit of our inclusion criteria, we extracted and analysed the appropriate data only. Studies had to target the general population (i.e., not specific sub-groups or minorities) and include at least one measure of obesity (e.g., body mass index (BMI)). The focus of this review is on the economic evaluation of behavioural interventions; economic analyses of pharmaceutical interventions, surgical interventions and structural interventions only were therefore excluded. Studies published in languages other than English or German were not considered for this review; this language restriction was mainly due to the authors’ language competencies, but it seems fair to assume that the vast majority of relevant articles are published in these languages.

### 2.3. Study Selection Procedure

In the first round, two reviewers (Nora Döring and Susanne Mayer) independently screened title and abstract. Uncertainties (i.e., at least one reviewer rated a study with “maybe”) or conflicting views about the inclusion of a specific paper were resolved by discussion or the final judgement of a third reviewer (Diana Sonntag). In the second round, the full texts of the remaining 11 articles were again independently assessed by two reviewers (Nora Döring; Susanne Mayer or Diana Sonntag) for their inclusion.

### 2.4. Data Extraction

An extraction form was developed and piloted on all six included studies by the authors (Nora Döring, Susanne Mayer and Diana Sonntag) and refined accordingly. The final extraction table included the following items: Article details (authors, year, title, reference), study characteristics (design, country, setting, underlying theoretical model), study population (target group, inclusion of parents (yes/no), analytical sample (*n*)), information on the intervention (mode of delivery, intervention components, control group, primary/secondary outcome measures), economic information (short description, cost categories, study perspective (assumed/reported), time horizon, discount rate, reference year, main results, sensitivity analysis). The final extraction was done by one author (Nora Döring) and checked for completeness and accuracy by two other authors (Susanne Mayer and Diana Sonntag).

### 2.5. Quality Assessment of Included Studies

Two researchers (Nora Döring and Susanne Mayer) independently applied the quality assessment checklist developed by the Centre for Reviews and Dissemination of the University of York [18], which is a slightly adapted version of the well-known Drummond checklist for economic evaluation. Given that none of the available checklists or scoring schemes had been validated to quantify the methodological quality, we did not apply quality assessment of individual items or an overall summary score. Instead, the quality assessment provides a systematic and critical descriptive overview of key methodological elements.

## 3. Results

**Figure 1:**
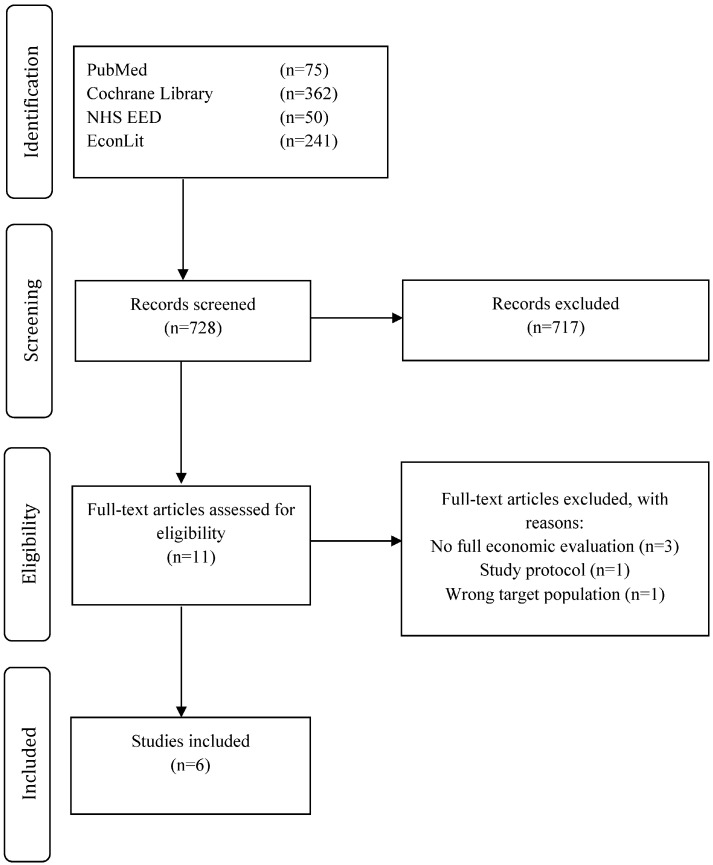
PRISMA (Preferred Reporting Items for Systematic Reviews and Meta-Analyses) flow diagram.

Figure 1 shows the flow of studies identified [20], screened and included in the review. Of the 728 studies identified in the initial search, 717 were excluded after screening based on titles and abstracts. Eleven articles remained for subsequent detailed assessment; of these, six were in line with eligibility criteria and were thus included in our analysis and synthesis. The main reasons for exclusion were the lack of (full) economic evaluation or not fulfilling inclusion criteria, e.g., children over six years of age. Although the study by Pil et al. [21] did not cover a full economic evaluation, it was included because it described the methods of a planned full economic evaluation.

### 3.1. General Characteristics

An overview of the general characteristics of the studies included is presented in Table S1 (see Supplementary Material). The majority of the studies (*n* = 4) originated from Australia, while one was from the United States and one was a multinational study from Europe. Because children’s ages ranged from early infancy to 12 years old, all intervention studies also included parents. Among the six studies considered were five intervention studies [21,22,23,24,25], of which three were randomised trials [22,24,25], one quasi-experimental intervention study [23] and one cross-European study [21]. The sixth study was based on a simulation approach including secondary data [26]. Studies were published between 2008 and 2014. Settings were diverse and included primary care [24,25], kindergarten [21], community [23] and the participants’ home [22].

### 3.2. Characteristics of the Intervention Programmes

All intervention studies included some elements of health promotion and focused on changes of behaviours associated with obesity (e.g., dietary habits, physical activity) delivered in consultations with parents. None of the studies reported a theoretical framework for the intervention programme. The intensity and duration of the intervention varied considerably in terms of number of sessions (ranging from three to eight) and intervention period (two weeks to two years). By contrast, the simulation study by Ma and Frick [26] was not based on a randomised controlled trial; instead, it estimated cost-savings of potential interventions that were targeted either to the paediatric population or to children with overweight and obesity.

None of the included studies restricted their sample to normal-weight children only. Some of them looked at only overweight/obese children [23,24,25,26] or a mixed-weight group [22,25]. As a result, these studies did not assess the cost-effectiveness of primary prevention when primary prevention is characterized by no symptoms of overweight or obesity. However, this is less surprising since the primary aim of a preventive intervention is to slow down rates of becoming overweight or prevent obesity and this can be achieved by both primary, secondary and tertiary prevention strategies. By virtue of our eligibility criteria, all studies had at least one type of obesity measure in their outcomes. Most commonly, these were age- and gender-specific BMI scores, based on objectively measured weight and height information. Two studies also included changes in health behaviour as a secondary outcome of the economic evaluation [21,25]. The comparison group was not always clearly specified but can be assumed to be care-as-usual in all of the intervention studies. While only one of the studies reported statistically significant effect sizes (i.e., a BMI unit reduction of 0.29 at age two) [22], the other described modest non-significant effect sizes. In the simulation study of Ma and Frick, different effect sizes were assumed [26]. In their methodological study, Pil et al. described how effects are expressed both in weight status and energy balance-related behaviours, which are then be extrapolated to weight status, obesity-related diseases and quality of life during adulthood [21].

### 3.3. Economic Findings

#### 3.3.1. Basic Characteristics

A summary of the economic findings, focusing on the primary outcomes of the economic evaluations as reported in the included studies, is shown in Tables S2 (A) and (B) (see Supplementary Material). All but one study aimed to assess the cost-effectiveness of a specific intervention, given the benefits and costs resulting from it. The studies by Moodie et al. [24] and Wake et al. [25] were based on the same intervention (i.e., LEAP, short for Live, Eat and Play); however, they differed in timing of follow-up and economic approach. In the simulation study by Ma and Frick [26], the question was turned around by examining the maximum intervention investment that can be made, given potential obesity-related costs.

One can differentiate between trial-based and model-based economic evaluation. The former examines costs and outcomes only within the observation time of the trial of interest, whereas a model-based evaluation goes beyond the observed period and extrapolates the costs and outcomes over a longer time horizon (e.g., lifetime). In addition, it supports the analysis with secondary data (e.g., morbidity and mortality data). This is especially relevant in the field of disease prevention and early childhood intervention, where the benefits may only be observed long after the trial is finished. Four of the included studies applied a model-based evaluation [21,23,24,26], while the other two relied on observed data [22,25]. Among the model-based studies, three of them [21,23,24] applied Markov model techniques whereas the fourth study [26] used econometric analysis/simulations (for further details see Tables S2 (A) and (B)). The use of these techniques comes, however, at a cost of relying on assumptions, e.g., maintenance of the intervention effect. Wake et al. emphasized in their study that they believe that the current epidemiological knowledge base is insufficient to allow meaningful modelling over the lifetime [25]. Indeed, all model-based studies assumed a maintenance of the intervention effect over lifetime [23,24,26] or at least until adulthood [21].

#### 3.3.2. Study Perspectives

An economic evaluation is of most importance to policy makers as it points out to whom the costs occur. Of the included studies, three were reported from a health care perspective [22,25,26] and three from a broader societal perspective [21,23,24]. Wake et al. [25] reported on having applied a health care perspective, but it can be assumed that they also applied a societal one because they included indirect costs (e.g., productivity losses due parents’ absence from work).

#### 3.3.3. Cost Components

Costs were categorised into programme delivery costs, direct medical costs (e.g., physician visits), direct non-medical costs (e.g., additional time and money required to meet changed dietary and physical activity practices) and indirect costs (e.g., productivity loss). The choice of inclusion differs substantially across studies. An overview of the cost categories included in each study is presented in Table S2 (A). In line with recommendations for cost-effectiveness analyses of interventions [27], the establishment costs (i.e., costs for development, material and scientific evaluations) were not considered in the respective studies [21,22,23,24,25]. Interestingly, the study by Wake et al. included the costs for additional time and money required to cover the changed dietary and physical activity practices [25], while Moodie et al. deliberately excluded these [23]. Considering that these expenses account, in the former case, for more than 90% of the costs associated with the intervention, one needs to be aware of the inclusion criteria of the costs when reporting and comparing cost-effectiveness results.

#### 3.3.4. Cost-Effectiveness Results

In three of the included studies the incremental cost-effectiveness ratio (ICER) is presented. In one of these studies [22], a natural unit (costs/BMI unit avoided) was used; the other two studies used a valued outcome, namely disability-adjusted life years (DALYs) [23,24], thus conducting a cost-utility analysis. In the cost-consequence analysis by Wake et al. [25] and the methodological paper by Pil et al. [21], the costs per participant are presented. In the simulation study by Ma and Frick [26], the outcome is presented as the maximum amount that can be spent for a 1% obesity reduction to break even, given the lifetime costs of obesity-related medical expenditures.

#### 3.3.5. Sensitivity Analysis

It is inevitable that an economic evaluation contains some degree of uncertainty in its assessment. In all studies at least one type of sensitivity analysis was conducted to assess the robustness of the results. The choice of sensitivity analysis may depend on the methodology applied or the setting in which the intervention was conducted. If, for example, one believes that the intervention setting may differ from the “real world”, one can test whether the overall results change by including values perceived as more realistic. Hayes et al. [22] changed the travel time of the nurses who performed the home visits, which substantially reduced the ICER from 4239 Australian Dollar to 2697 Australian Dollar per BMI unit avoided [22]. Model-based economic evaluations are often founded on strong assumptions, for example the long-term effect maintenance. Moodie et al. acknowledged the strong, most likely unrealistic assumption of full-effect maintenance and tested in a scenario analysis the consequences of reduced-effect maintenance, showing that the intervention remains cost-effective even if 80% of the intervention effect is lost [24]. For model-based economic evaluation, it has become common practice to apply probabilistic sensitivity analyses to assess the uncertainty around the parameters included.

#### 3.3.6. Decision-Making beyond Economic Results

In practice, economic and epidemiological information alone is not sufficient to make a decision on the implementation of a new intervention. In addition to their economic evaluation, Moodie et al. assessed their intervention in a second stage [24]. The ACE obesity working group established a list of filters that are relevant for policy makers and intervention providers. These include strength of evidence, feasibility of implementation, acceptability of stakeholders, sustainability and side effects.

### 3.4. Quality Assessment

None of the included studies fulfilled all of the quality criteria (Table S3, see Supplementary Material). However, most studies fulfilled a large number of these criteria. Furthermore, certain criteria were simply not applicable to each respective study (e.g., items 12–15 due to different perspectives chosen), while others were not reported. As a general pattern, the majority of studies lacked further specification, along with elaboration of their choices and assumptions. In addition, the description of model details and cost data was suboptimal in most of the studies, with Moodie et al. [23] being the exception.

## 4. Discussion

To the best of our knowledge, this is the first review to explore and analyze cost-effectiveness analyses of early childhood obesity prevention and therefore provides an important contribution to the current knowledge gap in a relevant and growing field. The primary aim of the present article was not to pool the available results of the cost-effectiveness of prevention programmes in early childhood to offer general conclusions. Given the differences in overweight and obesity prevalence, as well as dissimilar national health care systems, a translation of economic results from one to another national context seems challenging and of limited use [27]. Instead this review provides a systematic overview of methods and limitations, rounded off by our recommendations for designing more precise economic assessments. Despite a comprehensive and thorough search strategy, we were only able to include six relevant studies, which limits our analytical possibilities for this review. However, we were able to recognize three characteristics common to all included studies. First, the majority of studies assessed cost-effectiveness based on single interventions, which did not provide a statistically significant effect size. Still, most interventions were presented as cost-effective or even cost-saving. The insignificant or secondary effect sizes may weaken the validity of the respective conclusions. Second, the majority of studies chose a lifetime perspective. While this perspective clearly adheres to guidelines [28] and is especially relevant to studies in early lifetime, it relies on assumptions about, for example, the duration of treatment effect and the trajectories of obesity-related co-morbidities after end of trial. These assumptions on a trial’s effectiveness, especially on effectiveness maintenance, should be viewed with caution when within-trial evaluations show no significant intervention effect. Third, the reported ICERs differed substantially in magnitude (see Table S2 (B)). These differences result from heterogeneous modelling approaches, settings and study perspectives, including which costs have been measured and how they were monetarily evaluated. Due to heterogeneity in both application and methodology, a comparison of the economic findings and their translation into practice seems difficult and misleading.

Surprisingly, given the increasing number of well-conducted intervention studies in the field of early childhood obesity prevention [11,12,14,29], the number of published economic evaluations is small. Indeed, our review is based only on six studies, which itself confirms the limited research in this field. There are numerous reasons for this lack of economic evaluations. For example, Dietz and Gortmaker [30] argue mainly along the lines of the novelty of the research field and the methodological challenges associated with it. Nevertheless, we believe that an important reason is the lack of significant effect sizes. Indeed, despite an ever-growing number of interventions targeting early childhood obesity, only a few have proven to be (significantly) effective in preventing obesity, especially when looking at long-term effectiveness [12,14,30]. While all trial-based studies included in our review reported statistically non-significant effect sizes, economic evaluations may still be relevant as non-significant effect sizes cannot automatically be equated to no intervention effect [28]. Another reason why economic evaluations of ineffective intervention are of relevance is that one intervention may have a significantly lower cost compared to the other, thus making it a cost-reducing strategy. However, economic evaluations are often conducted post hoc, which makes it difficult to conduct a comprehensive cost-effectiveness analysis when all relevant data are not available. Retrospective economic evaluations tend to be imprecise since they rely on assumed and restored data and not the actual measured data during the intervention. It is therefore advisable to involve health economists during the design phase of an intervention. More importantly, the development of a toolbox including good-practice guidelines for intervention developers and evaluators would be helpful in order to collect relevant data alongside the trial. While effects of interventions are often captured in a standardized way, resource use data is often collected using bespoke (and usually non-standardised) resource use questionnaires which are difficult to compare with each other because of the heterogeneity of cost categories included in these questionnaires. Particularly, the indirect costs of overweight and obesity, such as those due to productivity losses, are not commonly collected alongside trials, which limits the scope of economic evaluations.

Nevertheless, even if data were collected simultaneously with the trial, they might be often insufficient to conduct meaningful economic evaluations for the following four reasons. Firstly, conventional approaches to evaluate cost-effectiveness may be of limited use. In their explorative review, Petrou and Gray already named a number of methodological challenges inherent in economic evaluations of early childhood intervention programmes in general [31]. Early childhood obesity intervention may pose even further challenges given the complexity of both the nature of childhood obesity and key obesogenic environments [32]. While the randomized-controlled trial (RCT) design provides the gold standard for evaluating interventions for effectiveness, it is, however, also of great relevance to provide a framework for economic evaluation that goes beyond evaluating trial-based interventions. To fully evaluate cost-effectiveness of other types of intervention designs, it may be advisable to go beyond individual health behaviour changes and incorporate other influences. Sonntag et al. [33] have shown, for example, that obesity-related dietary behaviour in early childhood is influenced not only by parents but also by five key obesogenic environments: schools, television, the Internet, retailers and promotional campaigns. An in-depth measurement of possible influences in intervention studies may provide a more comprehensive overview of the cost-effectiveness of early childhood interventions. Secondly, the economic and health benefits of early childhood intervention may, if ever, become only visible decades after the intervention has taken place. Therefore, one needs to extrapolate the effects observed during childhood to adulthood. However, there is so far no evidence that the effect attained during early childhood will be sustained [34]. Moreover, we still know too little about the independent effect of childhood obesity on co-morbidities of adult obesity and the so-called spill-over effects of interventions [32]. More specifically, interventions targeting early childhood obesity can also have positive effects on parents or siblings. Without incorporating these effects, one may possibly underestimate the cost-effectiveness. Thirdly, to facilitate comparisons across fields, cost-utility analyses are often preferred. However, it is currently challenging to measure quality of life in early childhood due to the lack of valid preference-based instruments for that age group. As a result, the evidence base around childhood obesity and health-related quality of life is weak but offers key challenges and opportunities for future research in this field. Indeed, for children aged five to six, no significant association was found between health-related quality of life and obesity status [6]. For the time being, with insufficient methodological support for health-related quality of life in early childhood, we recommend for trial-based economic evaluations to focus on using natural units (e.g., BMI units). In modelling studies, one could think of extrapolating the observed intervention effect on BMI to adult age and then the adult BMI to health-related quality of life based on secondary literature. Finally, many economic evaluations apply a societal perspective when assessing the cost-effectiveness of interventions. For example, losses in working productivity are commonly used in economic evaluations during adulthood. An adaption is necessary for childhood-specific characteristics, and it may thus be reasonable to include, e.g., costs due to loss of educational attainment [35].

## 5. Conclusions

While the number of childhood obesity intervention studies is steadily increasing, economic evaluations are rarely performed due to a regrettable lack of epidemiological data. In addition, existing evaluations are only of limited use for policy makers and intervention providers because they do not include a representative number of effects of interventions. We believe that an expanded conceptual approach to evaluate the cost-effectiveness of childhood obesity prevention programmes, together with a toolbox for efficient data acquisition, is urgently needed.

## Figures and Tables

**Table 1:** Study inclusion and exclusion criteria. ^1^

Inclusion Criteria	Exclusion Criteria
Trial-based cost-effectiveness analyses, simulation-based cost-effectiveness analyses	Reviews, meta analyses, qualitative studies, partial economic evaluations (i.e., only description of costs or outcomes), not peer reviewed
Target population: Preschool children (<6 years) and/or their parents	Selected target groups (e.g., low socioeconomic status, ethnic groups)
Intervention: Behavioural intervention targeting diet and physical activity	Pharmaceutical intervention, surgical intervention, structural intervention
Intervention outcome measures must include at least one of the following: BMI or waist circumference, overweight prevalence	
Language: English or German	No abstract available; conference abstracts
European countries, USA, Canada, Australia, New Zealand	Developing countries

^1^ As a part of the literature search, economic evaluation protocols and additional information related to included studies were checked.

## Supplementary Materials

The following are available online at www.mdpi.com/1660-4601/13/9/911/s1, Table S1: General characteristics of studies, Tables S2 (A) and (B): Main characteristics and primary outcomes of the economic evaluation, Table S3: Quality assessment.
