# Supplementary material for: Economic Evaluation of Obesity Prevention in Early Childhood: Methods, Limitations and Recommendations
Source: Int J Environ Res Public Health. 2016 Sep 13;13(9):911. doi: 10.3390/ijerph13090911 (PMC5036744; doi:10.3390/ijerph13090911)
Supplement: Supplementary file 1 [file ijerph-13-00911-s001.pdf]

# Supplementary Materials: Economic Evaluation of Obesity Prevention in Early Childhood: Methods, Limitations and Recommendations

**Nora Döring, Susanne Mayer, Finn Rasmussen and Diana Sonntag**

**Table S1.** General characteristics of studies.

| Authors | Year | Country | Design | Setting | Target Population | *N* (Analytical Sample) | Intervention Component | Under-Lying Theory | Mode of Delivery | Parents Included | Comparator | Primary Outcome | Effectiveness |
|---|---|---|---|---|---|---|---|---|---|---|---|---|---|
| Moodie et al. [1] | 2008 | Australia | RCT/CEA | Primary care | 5–9 years, overweight, mildly obese | 34 GPs and 163 children | Training of GP (3 times 2.5 h) 4 consultations over a 12-week period | Not reported | GP | Yes | Current practice | BMI | Not significant BMI mean difference = 0.25 (95% CI: −0.62, 0.12) |
| Wake et al. [2] | 2008 | Australia | RCT/CCA | Primary care | 5–9 years, overweight, mildly obese | 163 parents with children | Training of GP (3 times 2.5 h) 4 consultations over a 12-week period | Not reported | GP | Yes | Assumed current practice | BMI | Not significant BMI mean difference = −0.0 (−0.5, 0.5) |
| Ma and Frick [3] | 2011 | USA | Cohort study | n.a. | 0–6 years | 30,000,000 | n.a. | Not reported | n.a. | No | n.a. | BMI | n.a. |
| Moodie et al. [4] | 2013 | Australia | Quasi-experime ntal/CEA | Community setting | 4–12 years | 2184 children | Promoting healthy eating, physical activity and healthy weight gain with community drive and context-specific decision making | Not reported | Community service | Yes | A stratified random selection of preschools (*n* = 4) and primary schools (*n* = 12) | BMI | Not significant BMI mean difference = −0.28 (95% CI: −0.7, 0.15) |
| Hayes et al. [5] | 2014 | Australia | RCT/CEA | Home | Newborns | 324 parents with infants | 8 one-to-one consultations with age-appropriate education and advice on feeding, nutrition and physical activity | Not reported | Nurse | Yes | Care as usual, plus home safety information sent by mail | BMI | Not significant BMI mean difference = 0.33 (95% CI: −0.043, 0.662) |

| | | | | | | | | | | | | | |
|---|---|---|---|---|---|---|---|---|---|---|---|---|---|
| Pil et al. [6] | 2014 | Belgium Bulgaria, Germany, Greece, Poland, Spain | RCT/Methods for CEA | Kindergarten | 4–6 years | Simulation results are presented per 1000 | 3 training sessions for teachers | Not reported | Kinder-garten teacher | Yes | Not reported | Weight status | Not reported |

Notes: n.a. = not applicable; RCT = randomised controlled trial; CCA = cost-consequence analysis; CEA = cost-effectiveness analysis; CI = confidence interval; GP = general practitioner; BMI = body mass index.

**Table S2 (A).** Main characteristics and primary outcomes of the economic evaluation.

| Authors | Type | Model Specification | Study Perspective | Time Horizon | Cost Categories | Excluded Costs | Discount Rate | Year of Costing | Costs Per Child |
|---|---|---|---|---|---|---|---|---|---|
| Moodie et al. [1] | CEA | Cohort simulations Lifetime costs associated with obesity (ACE-approach) | Societal | Lifetime | Programme delivery costs Direct medical costs Indirect costs | Establishment costs | Costs: 3% Effects: 3% | 2001 | 650 AUD [a] |
| Wake et al. [2] | CCA | n.a. | Reported: Health care Assumed: Societal | Short (1 year) | Programme delivery costs Direct medical costs Direct non-medical costs Indirect costs | Establishment costs | n.a. | 2003 | 705 AUD/48 047 AUD [b] |
| Ma and Frick [3] | | econometric analysis/simulation Lifetime costs associated with obesity | Not reported Assumed: Health care | Lifetime | n.a. | n.a. | Costs: 3% Effects: not reported | 2006 | Up to 339 USD [c] |
| Moodie et al. [4] | CEA | Cohort simulations Lifetime costs associated with obesity (ACE-approach) | Societal | Lifetime | Programme delivery costs Direct non-medical costs Other costs | Student time, spin-off activities, cost associated with changes in the physical activity and eating patterns of participating families | Costs: 3% Effects: 3% | 2006 | 344 AUD |
| Hayes et al. [5] | CEA | n.a. | Health care | Short (2 years) | Programme delivery costs Direct medical costs Indirect costs | Costs associated with birth, evaluation or administration of the clinical trial | n.a. | 2012 | 1 309 AUD |

| | | | | | | | | | |
|---|---|---|---|---|---|---|---|---|---|
| Pil et al. [6] | CEA/ Design | Probabilistic Markov Model based on disease states | Societal | Lifetime | Programme delivery costs<br>Direct medical costs<br>Indirect costs | Establishment costs | Costs: 3%<br>Effects: 1.5% | 2012 | Belgium: 11.24 EUR<br>Bulgaria: 6.07 EUR<br>Germany: 28.84 EUR<br>Poland: 5.25 EUR<br>Spain: 8.94 EUR [d] |

Notes: n.a. = not applicable; BMI = body mass index; CCA = cost-consequence analysis; CEA = cost-effectiveness analysis; DALY = disability adjusted life year; GP = general practitioner; BMI = body mass index; PSA = probabilistic sensitivity analysis. [a] Based on own calculation: the article presents total costs: 6,300,000 AUD, 9685 children assumed to be reached. [b] Two different numbers are mentioned. 705 AUD refers to the costs per child for the health care sector, 48,047 AUD refers to the total cost per child including productivity losses and direct non-medical costs (i.e., expenditure due to changed physical activity and eating habits). [c] Based on simulation analysis, maximum amount that could be spent per child for 1 BMI unit reduction for the age group 0–6 in a population-based approach. [d] Based on own calculation, the article presents an estimation of costs for 1000 pre-schoolers.

**Table S2 (B).** Main characteristics and primary outcomes of the economic evaluation.

| ICER | Uncertainly Analysis | Sensitivity Analysis | Cost-Effective |
|---|---|---|---|
| 4680 AUD/DALY | PSA | Full maintenance of BMI effects into adulthood/vs. half maintenance,<br>Recruitment rates,<br>Family attendance<br>Delivery of intervention,<br>Outlier removal | Yes |
| n.a. | - | Value of parents' time<br>Unit cost of GP<br>Economies of scale<br>combinations | No |
| n.a. | - | Excluding obesity-related medical costs after certain ages | n.a. |
| 29,798 AUD/DALY | PSA | | Yes |
| 4320 AUD/BMI | - | Adjustments in travel time | Yes |
| n.a. | (PSA) | | n.a. |

**Table S3.** Quality assessment.

| Item | | Moodie et al. (2008) [1] | Wake et al. (2008) [2] | Ma and Frick (2011) [3] | Moodie et al. (2013) [4] | Hayes et al. (2014) [5] | Pil et al. (2014) [6] |
|---|---|---|---|---|---|---|---|
| Study design | | | | | | | |
| 1 | Research question | ✓ | ✓ | ✓ | ✓ | ✓ | ✓ |
| 2 | Economic importance | ✓ | ✓ | ✓ | ✓ | ✓ | ✓ |
| 3 | Viewpoint | ✓ | ✓ | ✓ | ✓ | ✓ | ✓ |
| 4 | Rationale for choice of comparator | ✓ | | n.a. | ✓ | ✓ | |
| 5 | Description of comparator | ✓ | | n.a. | ✓ | ✓ | |
| 6 | Form of economic evaluation | ✓ | ✓ | | ✓ | ✓ | ✓ |
| 7 | Justification of form | ✓ | ✓ | | ✓ | ✓ | ✓ |
| Data collection | | | | | | | |
| 8 | Sources of effectiveness estimates | ✓ | ✓ | ✓ | ✓ | ✓ | n.a. |
| 9 | Design and results of effectiveness study | ✓ | ✓ | ✓ | ✓ | ✓ | n.a. |
| 10 | Details and results of meta-analysis | n.a. | n.a. | ✓ | n.a. | n.a. | n.a. |
| 11 | Primary outcome | ✓ | ✓ | ✓ | ✓ | ✓ | ✓ |
| 12 | Methods to value health states | ✓ | n.a. | n.a. | ✓ | n.a. | ✓ |
| 13 | Subjects who valued the health states | ✓ | n.a. | n.a. | ✓ | n.a. | ✓ |
| 14 | Productivity changes | ✓ | n.a. | n.a. | ✓ | n.a. | ✓ |
| 15 | Relevance of productivity changes | | n.a. | n.a. | ✓ | n.a. | ✓ |
| 16 | Separate reporting of costs | ✓ * | ✓ * | n.a. | ✓ * | ✓ * | ✓ * |
| 17 | Methods of estimation of costs | ✓ | ✓ | ✓ | ✓ | ✓ | ✓ |
| 18 | Currency and price data | ✓ | ✓ | ✓ | ✓ | ✓ | ✓ |
| 19 | Price adjustment for inflation | ✓ | n.a. | ✓ | ✓ | ✓ | ✓ |
| 20 | Model details | ✓ | | ✓ | ✓ | | ✓ |
| 21 | Justification of model choice | | | ✓ | ✓ | | ✓ |
| Analysis and interpretation of results | | | | | | | |
| 22 | Time horizon | ✓ | ✓ | ✓ | ✓ | ✓ | ✓ |
| 23 | Discount rate | ✓ | n.a. | ✓ | ✓ | ✓ | ✓ |
| 24 | Justification of choice | ✓ | n.a. | | ✓ | ✓ | ✓ |
| 25 | Explanation for absence of discount rate | n.a. | n.a. | | ✓ | n.a. | n.a. |
| 26 | Details for statistical tests (incl. CI) | ✓ | | | ✓ | ✓ | |
| 27 | Sensitivity analysis | ✓ | ✓ | ✓ | ✓ | ✓ | ✓ |
| 28 | Justification for sensitivity analysis | ✓ | ✓ | ✓ | ✓ | ✓ | ✓ |
| 29 | Parameter ranges | ✓ | | | ✓ | ✓ | |

| | | | | | | | |
|---|---|---|---|---|---|---|---|
| 30 | Relevant comparison | ✓ | ✓ | n.a. | ✓ | ✓ | |
| 31 | Incremental analysis | ✓ | | | ✓ | ✓ | |
| 32 | Disaggregated and aggregated | ✓ | n.a. | ✓ | ✓ | ✓ | |
| 33 | Answer to study question | ✓ | ✓ | ✓ | ✓ | ✓ | ✓ |
| 34 | Conclusion | ✓ | ✓ | ✓ | ✓ | ✓ | ✓ |
| 35 | Caveats around conclusion | ✓ | ✓ | ✓ | ✓ | ✓ | ✓ |
| 36 | Generalisability | | ✓ | | ✓ | ✓ | ✓ |

Notes: CI = confidence interval. Quality assessment based on Jo Akers, Raquel Aguiar-Ibáñez, Ali Baba-Akbari Sari, Susanne Beyon, Alison Booth, Jane Burch, Duncan Chambers et al. CRD's Guidance for Undertaking Reviews in Health Care. York (UK): Centre for Reviews and Dissemination (CRD). 2009. Detailed information about the assessment of quality indicators can be received upon author request. * categorized reporting of cost. Quantities not reported separately from cost.

## References


1. Moodie, M.; Haby, M.; Wake, M.; Gold, L.; Carter, R. Cost-effectiveness of a family-based GP-mediated intervention targeting overweight and moderately obese children. *Econ. Hum. Biol.* **2008**, *6*, 363–376.
2. Wake, M.; Gold, L.; McCallum, Z.; Gerner, B.; Waters, E. Economic evaluation of a primary care trial to reduce weight gain in overweight/obese children: The LEAP trial. *Ambul. Pediatr.* **2008**, *8*, 336–341.
3. Ma, S.; Frick, K.D. A simulation of affordability and effectiveness of childhood obesity interventions. *Acad. Pediatr*. **2011**, *11*, 342–350.
4. Moodie, M.L.; Herbert, J.K.; de Silva-Sanigorski, A.M.; Mavoa, H.M.; Keating, C.L.; Carter, R.C.; Waters, E.; Gibbs, L.; Swinburn, B.A. The cost-effectiveness of a successful community-based obesity prevention program: The be active eat well program. *Obesity* **2013**, *21*, 2072–2080.
5. Hayes, A.; Lung, T.; Wen, L.M.; Baur, L.; Rissel, C.; Howard, K. Economic evaluation of "healthy beginnings" an early childhood intervention to prevent obesity. *Obesity* **2014**, *22*, 1709–1715.
6. Pil, L.; Putman, K.; Cardon, G.; De Bourdeaudhuij, I.; Manios, Y.; Androutsos, O.; Lateva, M.; Lotova, V.; Zych, K.; Góźdź, M.; et al. Establishing a method to estimate the cost-effectiveness of a kindergarten-based, family-involved intervention to prevent obesity in early childhood. *Obes. Rev.* **2014**, *15*, 81–89.


© 2016 by the authors; licensee MDPI, Basel, Switzerland. This article is an open access article distributed under the terms and conditions of the Creative Commons by Attribution (CC-BY) license (http://creativecommons.org/licenses/by/4.0/).
